# Optimal exercise interventions for enhancing cognitive function in older adults: a network meta-analysis

**DOI:** 10.3389/fnagi.2025.1510773

**Published:** 2025-07-11

**Authors:** Han Han, Jinhao Zhang, Fan Zhang, Fanghui Li, Zhijian Wu

**Affiliations:** ^1^Department of Police Physical Education, Jiangsu Police Institute, Nanjing, China; ^2^School of Sport Sciences, Nanjing Normal University, Nanjing, China; ^3^Special Police Academy, Nanjing Police University, Nanjing, China

**Keywords:** exercise, cognitive function, network meta-analysis, older adults, resistance training, mind-body exercise, aerobic exercise, executive function

## Abstract

**Background:** Cognitive decline poses a significant challenge to healthy aging. While exercise is widely recognized for its cognitive benefits, the comparative efficacy of different exercise modalities and optimal intervention protocols for specific cognitive domains in older adults remains unclear.

**Objective:** This network meta-analysis aimed to systematically compare the effects of five exercise modalities—resistance training, aerobic exercise, mind-body exercise, multicomponent exercise, and high-intensity interval training (HIIT)—on global cognitive function and major cognitive domains in cognitively healthy older adults, and to identify optimal intervention protocols and population subgroups most likely to benefit.

**Methods:** A total of 58 randomized controlled trials (RCTs) were included, encompassing 4,349 healthy older adults from diverse geographical regions. Comprehensive searches were conducted in major electronic databases for RCTs evaluating exercise interventions on cognitive outcomes in adults aged 60 years and older. A network meta-analysis assessed the relative effects of each exercise modality on global cognition, executive function (including inhibitory control, task-switching ability, and working memory), and memory function. Subgroup analyses were performed based on intervention frequency, duration, participant age, and geographic region.

**Results:** Resistance training demonstrated the greatest improvement in global cognitive function (SMD = 0.55) and inhibitory control (SMD = 0.31, SUCRA = 82.1%), particularly with twice-weekly sessions of 45 min over 12 weeks. Mind-body exercise was most effective for executive function, especially task-switching ability (SMD = −0.58, SUCRA = 85.1%) and working memory (SMD = 2.45), with high-frequency, moderate-duration protocols yielding optimal results. Aerobic exercise was the most effective modality for enhancing memory function (SMD = 0.42). The largest cognitive benefits were observed in participants aged 65–75 years and in studies conducted in Asia.

**Conclusion:** Different exercise modalities provide domain-specific cognitive benefits in healthy older adults. Personalized exercise prescriptions—emphasizing resistance training for global cognition, mind-body exercise for executive function, and aerobic exercise for memory—should be considered in clinical and public health settings. These findings support the integration of structured exercise interventions into aging and dementia prevention strategies, with particular attention to optimal protocol design and population targeting.

## 1 Introduction

With increasing life expectancy and declining birth rates worldwide, the proportion of older adults is growing rapidly, presenting critical challenges to public health systems. Cognitive decline, even in the absence of clinical dementia, can lead to diminished quality of life, impaired independence, and greater risk for institutionalization (Livingston et al., 2020). While pharmacological treatments for cognitive impairment remain limited in efficacy and carry potential adverse effects, physical exercise has emerged as a promising non-pharmacological intervention for promoting brain health (Northey et al., 2018). Numerous randomized controlled trials (RCTs) and meta-analyses have demonstrated the positive effects of physical activity on global cognition in older adults (Sofi et al., 2011). Proposed mechanisms include enhanced cerebral blood flow, increased expression of neurotrophic factors such as brain-derived neurotrophic factor (BDNF), reduced systemic inflammation, and improved neurovascular coupling (Kirk-Sanchez and McGough, 2014). Despite this growing body of evidence, several knowledge gaps remain. First, most prior studies treat cognition as a single composite construct, neglecting its domain-specific organization. Cognitive functioning in older adults encompasses multiple subdomains—such as inhibitory control, task switching, working memory, episodic memory, verbal fluency, and attention—each of which may respond differently to various types of exercise (Gheysen et al., 2018). For example, resistance training has been linked to improved executive function, whereas mind–body practices such as Tai Chi may offer greater benefits for memory and attention (Zhang et al., 2023). Second, intervention efficacy may be modulated by exercise dosage, including frequency, session duration, and total program length. However, these parameters are often inconsistently reported or analyzed across studies, limiting the ability to recommend optimized protocols (Liu-Ambrose et al., 2010; Falck et al., 2019). In addition, regional and cultural factors may influence the acceptability and efficacy of certain exercise types, especially in non-Western populations where mind–body exercises are more commonly practiced (Cheng et al., 2016). To address these gaps, we conducted an updated and comprehensive meta-analysis integrating network and pairwise comparisons based on 58 unique RCTs involving 3,943 cognitively healthy older adults. This study aims to (1) evaluate and rank the effects of different exercise interventions on both global and domain-specific cognitive outcomes; (2) investigate the moderating role of intervention parameters including frequency, session length, and duration; and (3) offer evidence-based recommendations for domain-specific and culturally tailored exercise prescriptions in aging populations.

## 2 Methods

This systematic review and meta-analysis were conducted in accordance with the Cochrane Handbook for Systematic Reviews of Interventions (Higgins et al., 2019) and followed the PRISMA (Preferred Reporting Items for Systematic Reviews and Meta-Analyses) guidelines to ensure transparent and reproducible reporting (Moher et al., 2009).

### 2.1 Literature search

We systematically searched PubMed, Web of Science, ScienceDirect, CNKI, and Wanfang Data for randomized controlled trials (RCTs) evaluating the effects of exercise interventions on cognitive function in healthy older adults. Search terms included combinations of “exercise” OR “physical activity,” AND “cognitive function” OR “cognition,” AND “older adults” OR “elderly,” AND “memory” OR “executive function,” AND “randomized controlled trial” OR “RCT,” AND “healthy aging.” Searches included both English and Chinese publications and covered all articles published before January 1, 2024. Additionally, reference lists from relevant systematic reviews and meta-analyses were manually screened to identify additional studies (Gallardo-Gomez et al., 2022; Gavelin et al., 2021). The complete search strategy is available in Supplementary Material 1.

### 2.2 Inclusion and exclusion criteria

Inclusion Criteria: (1) The participants were healthy older adults, mean aged 55 years and above, regardless of gender or nationality, without a diagnosis of cognitive impairment or dementia. (2) The exercise interventions in this study were classified into five main types based on their assumed physiological mechanisms. This classification was made to reflect the different effects each type of exercise may have on cognitive function in older adults. The exercise types and their rationale for classification are as follows: Resistance Training (RES), this type includes exercises that primarily aim to improve muscle strength and endurance through controlled movements against resistance (e.g., weightlifting, using resistance bands). Resistance training is known for its positive impact on brain-derived neurotrophic factor (BDNF), which supports neuroplasticity and cognitive functions such as inhibitory control and executive function. It is classified separately due to its emphasis on building muscular strength and the distinct physiological responses it triggers compared to aerobic exercise. Aerobic exercise (AER), aerobic exercise involves activities that increase cardiovascular fitness through sustained, rhythmic exercises such as walking, running, cycling, or swimming. The focus of aerobic exercise is on improving heart and lung function. It is classified as a distinct category because it has been shown to enhance memory, hippocampal neurogenesis, and cerebral blood flow, which directly contribute to improvements in memory and other cognitive functions in older adults. High-Intensity Interval Training (HIIT), HIIT consists of alternating between short bursts of intense activity followed by periods of lower-intensity recovery. This form of exercise is classified separately due to its unique approach, focusing on intense, time-efficient bouts of physical activity. HIIT has been shown to improve cardiovascular health, metabolic efficiency, and cognitive function, particularly in areas related to executive control and processing speed. Physical and Mental Training (PMT), this category encompasses exercises that require both physical movement and mental focus, such as Tai Chi, yoga, and other mind-body practices. These exercises are classified separately because they engage both physical and cognitive aspects simultaneously, promoting relaxation, stress reduction, and improvements in executive functions such as task-switching and working memory. This classification reflects the holistic nature of these interventions, which are designed to improve both physical balance and cognitive flexibility. Multi-modal Exercise (CEX), multi-modal exercise interventions combine different forms of exercise (e.g., a mix of aerobic, resistance, and flexibility training). The classification as multi-modal exercise is based on the premise that combining multiple exercise types may provide broader cognitive benefits by engaging different physiological systems. This comprehensive approach is intended to enhance multiple aspects of physical fitness and cognitive health simultaneously. This classification aligns with the World Health Organization (WHO) exercise guidelines, which categorize exercise types based on their physiological and functional outcomes. The goal of this classification was to examine the distinct cognitive effects of each exercise modality, as they are thought to influence various cognitive domains differently, such as memory, executive function, and overall cognitive health. (3) Control groups received either health education or maintained their usual activities. (4) The primary outcome of this study was overall cognitive function, which was assessed using the Montreal Cognitive Assessment (MoCA) and the Mini-Mental State Examination (MMSE). Secondary outcomes focused on specific aspects of executive function and memory, including: Inhibitory Control, assessed using the Stroop Test (measuring response inhibition to conflicting stimuli) and the Go/No-Go Task (evaluating suppression of prepotent responses). Task-Switching Ability, measured through the Trail Making Test Part B (assessing cognitive flexibility between alphanumeric sets) and the Wisconsin Card Sorting Test (evaluating rule-based switching and adaptability). Working Memory, evaluated using the N-back Test (spatial and verbal updating) and the Backward Corsi Block-Tapping Task (visuospatial manipulation and retention). Memory Function, assessed with Logical Memory Recall (short-and long-term episodic memory) and the Rey Auditory Verbal Learning Test (multitribal verbal encoding and retrieval). These secondary outcomes were selected to provide a deeper understanding of how different exercise modalities impact various aspects of cognitive function beyond overall cognition. (5) The included studies were randomized controlled trials (RCTS) to ensure high-quality evidence.

Exclusion Criteria: (1) Studies with duplicate publications. (2) Studies lacking extractable outcome measures or reporting incomplete data. (3) Non-RCTs or trials with inadequate control group comparisons.

### 2.3 Study selection

After the initial search, all identified articles were imported into NoteExpress reference management software to remove duplicates. Two independent reviewers screened the titles and abstracts based on the pre-defined inclusion and exclusion criteria. Studies that did not meet the criteria were excluded, and any disagreements between reviewers were resolved by a third-party consensus (Markov et al., 2023). Full-text articles of the remaining studies were retrieved and further evaluated for eligibility through a second screening.

### 2.4 Data extraction

Data were extracted by two independent reviewers using a standardized data extraction form based on the Cochrane Handbook for Systematic Reviews guidelines. The extracted data included: Study Characteristics: Title, first author, publication year, study design. Participant Characteristics: Sample size, mean age, gender distribution. Intervention Details: Type, duration, frequency, intensity of exercise intervention, and control group details. Outcome Measures: Pre- and post-intervention data for cognitive function and specific executive functions. To ensure data accuracy, all extracted data were cross-verified by the two reviewers, with discrepancies resolved through consensus or consultation with a third reviewer.

### 2.5 Quality assessment

The risk of bias in the included studies was assessed independently by two reviewers using the Cochrane Collaboration’s Risk of Bias Tool (Courel-Ibanez et al., 2019). This tool evaluates potential biases across seven domains: random sequence generation, allocation concealment, blinding of participants and personnel, blinding of outcome assessment, incomplete outcome data, selective reporting, and other biases. Studies were classified into three categories based on their risk of bias: Low risk: 4 or more domains with low risk. Moderate risk: 2–3 domains with low risk. High risk: 1 or fewer domains with low risk. Additionally, publication bias was assessed using funnel plots and Egger’s test, with statistical significance set at *p* < 0.05 (Egger et al., 1997).

### 2.6 Statistical analysis

All statistical analyses were conducted using Review Manager 5.3 and Stata 17.0 software. The standardized mean differences (SMD) were calculated using Cohen’s d for studies with larger sample sizes, and Hedge’s g was used for studies with smaller sample sizes to correct for potential bias. Specifically: Cohen’s *d* was computed using the formula: *d* = (M1–M2)/SD pooled. where M1 and M2 represent the means of the experimental and control groups, respectively, and SD pooled is the pooled standard deviation. Hedge’s g was applied for studies with smaller sample sizes using the formula: $g=\frac{M_{1}-M_{2}}{{\text{SD}}_{p\,o\,o\,l\,e\,d}}\times \left(1-\frac{3}{4\,\left(N_{1}+N_{2}\right)-9}\right)$ where N1 and N2 are the sample sizes of the experimental and control groups, respectively. For studies reporting data in non-standard formats, appropriate formulas were used to convert the data into mean and standard deviations (SD) (Higgins et al., 2019).

To evaluate network transitivity, we compared the clinical and methodological characteristics of studies to ensure that the multiple treatment comparisons were adequately comparable. The inconsistency and consistency models were tested using both the design-by-treatment interaction model (a global approach) and the node-splitting test (a local approach). The node-splitting test was used to identify any substantial discrepancies between direct and indirect comparisons for each treatment, evaluating the consistency of the results.

The probability values were compiled and presented as the surface under the cumulative ranking curve. The exercise interventions were ranked by using the surface under the cumulative ranking curve and mean rank. Model fit was assessed using the Deviance Information Criterion (DIC), where a lower DIC value indicated a better model fit. To evaluate consistency in the network, node-splitting analysis was conducted to compare direct and indirect estimates, with a *p*-value > 0.05 indicating no significant inconsistency. Additionally, heterogeneity across studies was assessed using the *I*^2^ statistic, with *I*^2^ > 50% considered indicative of substantial heterogeneity. To determine the ranking of exercise interventions, we used surface under the cumulative ranking curve (SUCRA) values. A SUCRA value close to 100% indicates a high probability of being the most effective intervention, whereas a value close to 0% suggests the least effective intervention. Interventions were ranked based on their mean SUCRA scores, allowing a probabilistic ranking of effectiveness across cognitive domains.

The analysis focused on five key indicators of cognitive function: overall cognitive function, inhibitory control, task-switching ability, working memory, and memory. Heterogeneity among studies was assessed using the *I*^2^ statistic, where an *I*^2^ value above 50% indicated substantial heterogeneity, and a random-effects model was applied. Otherwise, a fixed-effects model was used (Talar et al., 2022). A network meta-analysis was conducted to compare the relative effectiveness of different exercise modalities, allowing for both direct and indirect comparisons across studies (Wei et al., 2022) .

## 3 Results

### 3.1 Study selection

**FIGURE 1:**
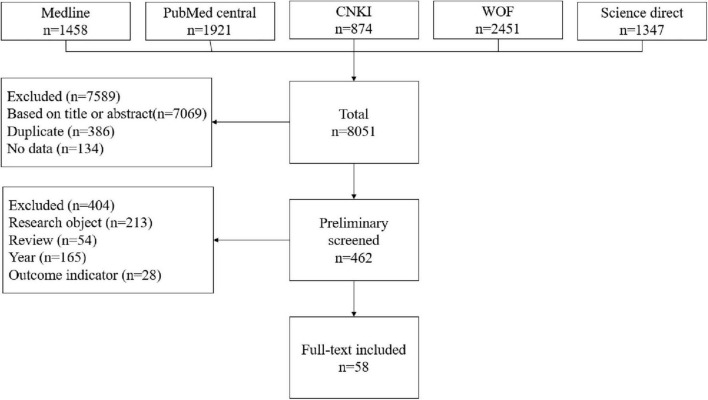
Study selection.

A total of 8,051 articles were retrieved from databases. After removing duplicates, 7,665 articles proceeded to the screening phase. Titles and abstracts were reviewed, resulting in the exclusion of 7,069 articles unrelated to the research topic, leaving 596 articles that potentially met the inclusion criteria. Subsequently, the full texts of these articles were further examined for detailed screening, leading to the exclusion of 538 articles that did not meet the inclusion criteria (Research object, *n* = 213; Review, *n* = 54; Year, *n* = 165; Outcome indicator, *n* = 106). Finally, 58 randomized controlled trials were included. The flow diagram in Figure 1 illustrates the detailed process for each stage.

### 3.2 Characteristics of included studies

A total of 58 randomized controlled trials (RCTs) were included in this meta-analysis, encompassing 4,349 healthy older adults from diverse geographical regions, including Asia (China, Japan, Vietnam), Europe (Italy, France, Spain, Portugal, Sweden, Netherlands), North America (USA, Canada), South America (Brazil, Colombia), Africa (South Africa), and the Middle East (Tunisia). The average age of participants ranged from 60 to 83.7 years, with most studies targeting individuals over 65 years old. The studies involved a wide variety of exercise modalities, which were categorized into five main types according to their physiological characteristics and cognitive engagement: Aerobic exercise (AER), Resistance training (RES), High-intensity interval training (HIIT), Physical and mental training (PMT)—including Tai Chi and yoga, and Multi-modal training (MMT) that combines two or more types of interventions. AER was the most frequently applied intervention across studies, followed by PMT and RES. The intervention duration ranged from 4 to 65 weeks, with session lengths between 30 and 90 min, and frequencies from 1 to 5 sessions per week. Most interventions lasted for at least 12 weeks, ensuring sufficient exposure for cognitive effects.

The control conditions varied across studies: 22 trials employed no exercise or waitlist controls, 13 used health education, 10 adopted stretching or balance training, and others involved minimal physical activity. The diversity of control conditions ensured a broad spectrum of comparison standards for evaluating intervention effects. As for outcome assessment, the studies examined a variety of cognitive domains, including: Overall cognitive function (MoCA, MMSE), Inhibitory control (e.g., Stroop test, Go/No-Go task), Task switching (e.g., TMT-B, Wisconsin Card Sorting Test), Working memory (e.g., n-back task, backward digit span), Verbal and episodic memory (e.g., logical memory test, RAVLT). These outcome measures allowed for a detailed analysis of the differential impact of exercise modalities on specific cognitive functions in aging populations. Detailed characteristics of the included studies are presented in Table 1 (Albinet et al., 2016; Ansai et al., 2015; Baklouti et al., 2023; Brown et al., 2009; Carral et al., 2007; Cassilhas et al., 2007; Castaño et al., 2022; Chen et al., 2014; Chen et al., 2020; Coelho et al., 2012; Coetsee and Terblanche, 2017; Colcombe and Kramer, 2003; Courel-Ibanez et al., 2019; Egger et al., 1997; Engeroff et al., 2022; Fabre et al., 2002; Farinha et al., 2021; Gallardo-Gomez et al., 2022; García-Garro et al., 2020; Gavelin et al., 2021; Gheysen et al., 2018; Gothe et al., 2017; Gu et al., 2019; Guo et al., 2024; Hanley et al., 2010; Higgins et al., 2019; Huang et al., 2022; Iuliano et al., 2015; Jiang et al., 2020; Jonasson et al., 2016; Coelho-Júnior et al., 2020; Kimura et al., 2010; Kirk-Sanchez and McGough, 2014; Kovacevic et al., 2020; Lachman et al., 2006; Lavretsky et al., 2011; Legault et al., 2011; Li, 2019; Lim et al., 2024; Liu et al., 2018; Liu, 2021; Liu D. M. et al., 2023; Jiang, 2020; Li et al., 2022; Liu-Ambrose et al., 2010; Livingston et al., 2020; Loprinzi et al., 2019; Lu et al., 2016; Maki et al., 2012; Markov et al., 2023; Miyazaki et al., 2022; Moher et al., 2009; Moreira et al., 2018; Mortimer et al., 2012; Muscari et al., 2010; Nagamatsu et al., 2012; Nguyen and Kruse, 2012; Nishiguchi et al., 2015; Northey et al., 2018; Norton et al., 2014; Oken et al., 2006; Peng, 2021; Petersen et al., 2014a; Predovan et al., 2012; Riske et al., 2017; Schley et al., 2006; Smith et al., 2010; Sofi et al., 2011; Sun et al., 2011; Sun et al., 2015; Sun, 2021; Talar et al., 2022; Taylor-Piliae et al., 2010; Timmons et al., 2018; Tsai et al., 2017; van de Rest et al., 2014; Varela et al., 2018; Vaughan et al., 2014; Venturelli et al., 2010; Wei et al., 2022; Williamson et al., 2009; Witte et al., 2016; Xu, 2022; Yang et al., 2020; Zhang et al., 2022, 2023; Zheng et al., 2023).

**TABLE 1:** Included in the study characteristics table.

References	Country	Sample size	Age (years)	Exercise type	Duration (min)	Frequency	Weeks	Control group	Outcome measures
Vizioli et al., 2009	Italy	120	69.2 ± 2.7	AER	60	3/w	52	Health education	①
Kovacevic et al., 2020	Canada	61	72.0 ± 5.7	HIIT/AER	28/47	3/w	12	No exercise	②⑤
Oken et al., 2006	USA	135	72.1 ± 4.9	PMT/AER	90/60	1/w	26	No exercise	②⑤⑦
Brown et al., 2009	Australia	120	79.6 ± 6.3	RES	60	2/w	25	No exercise	②③⑤
Fabre et al., 2002	France	16	65.6 ± 1.8	AER	60	2/w	8	No exercise	⑤
Coetsee and Terblanche, 2017	South Africa	67	62.7 ± 5.7	RES/HIIT/AER	30/30/47	3/w	16	No exercise	②
Farinha et al., 2021	Portugal	74	72.4 ± 5.1	AER/MMT	45/45	2/w	28	No exercise	①
Castaño et al., 2022	Brazil	12	70.0 ± 8.1	RES	60	2/w	16	Not arranged	③⑤⑥
Hanley et al., 2010	USA	32	72.9 ± 9.3	RES	60	2/w	4	No exercise	②③⑤
Albinet et al., 2016	France	36	60–75	AER	60	2/w	21	Stretching	②④⑤
Legault et al., 2011	USA	36	76.5 ± 4.9	AER	75	2/w	16	No exercise	②④⑤
Predovan et al., 2012	Canada	50	67.9 ± 6.2	AER	60	3/w	12	No exercise	②
Iuliano et al., 2015	Italy	60	66.9 ± 11.7	RES/HIIT	30/30	1/w	12	No exercise	②③
Coelho-Júnior et al., 2020	Brazil	26	66.7 ± 4.7	RES	60	2/w	24	No exercise	①⑤
Carral et al., 2007	Spain	62	68.4 ± 3.4	RES/AER	45/45	2/w	21	Not arranged	①
Mortimer et al., 2012	USA	90	67.8 ± 5.6	PMT/AER	50/50	3/w	40	No exercise	①②③⑤⑥⑦
Timmons et al., 2018	Ireland	84	69.3 ± 3.5	AER/RES/MMT	24/24/24	3/w	12	No exercise	①
Williamson et al., 2009	USA	102	77.4 ± 4.3	MMT	50	3/w	52	Health education	①②⑤
Sun et al., 2015	China	138	69.2 ± 5.9	PMT	60	2/w	25	No exercise	①
Ansai et al., 2015	Brazil	69	82.4 ± 2.4	MMT/RES	60/NR	3/w	16	No exercise	①⑥
Kimura et al., 2010	Japan	119	74.3 ± 5.5	RES	90	2/w	12	Health education	③
Jonasson et al., 2017	Sweden	58	68.7 ± 2.7	AER	45	3/w	26	Stretching	③④⑤
Lavretsky et al., 2011	USA	73	70.6 ± 7.3	PMT	50	2/w	10	No exercise	①⑤
Nguyen and Kruse, 2012	Germany	96	68.9 ± 5.1	PMT	60	2/w	26	No exercise	③
Lachman et al., 2006	USA	210	74.9 ± 6.9	RES	35	3/w	26	No exercise	⑤
Venturelli et al., 2010	Italy	23	83.7 ± 6.2	RES	45	3/w	12	No exercise	①
Nagamatsu et al., 2012	Canada	86	74.5 ± 3.5	RES/AER	60	2/w	26	Balance training	①
Moreira, 2017	Brazil	45	83.6 ± 3.9	MMT	50	3/w	16	No exercise	①
Gothe et al., 2017	USA	108	62.1 ± 5.6	PMT	60	3/w	8	Stretching	③⑤
Cassilhas et al., 2007	Colombia	43	67.7 ± 0.9	RES	60	3/w	24	No exercise	⑤⑦
Nishiguchi et al., 2015	Japan	48	73.3 ± 5.1	MMT	90	1/w	12	No exercise	①③⑤
Smiley, 2008	USA	57	70.2 ± 4.5	AER	50	3/w	42	Stretching	②
Vaughan et al., 2014	Australia	49	68.8 ± 3.3	MMT	60	2/w	16	No exercise	②③⑤
Taylor-Piliae et al., 2010	USA	93	69.2 ± 6.2	PMT/MMT	45/45	4/w	25	No exercise	⑤⑥
Liu-Ambrose et al., 2010	Canada	88	69.6 ± 2.9	RES	60	2/w	52	Stretching	②③⑤
Tsai et al., 2017	China	42	66.3 ± 4.4	AER	40	3/w	24	Balance training	①
van de Rest et al., 2014	Netherlands	58	77.8 ± 8.4	RES	30	2/w	24	No exercise	②③⑤⑥⑦
Varela et al., 2018	Spain	39	81.1 ± 8.2	AER	15	7/w	65	No exercise	①⑤
Witte et al., 2016	Germany	54	68.4 ± 4.6	AER	60	2/w	21	No exercise	①
Maki et al., 2012	Japan	150	72.0 ± 4.0	AER	90	1/w	13	Health education	③⑤⑥
Chen et al., 2020	China	29	62.4 ± 3.1	PMT/AER	90/90	5/w	24	Not arranged	③
Chen et al., 2014	China	125	66 ± 11.8	AER	45	4/w	52	No exercise	①
Jiang et al., 2020	China	30	64.0 ± 3.7	PMT	60	3/w	12	No exercise	②③④
Li, 2019	China	74	66.3 ± 4.5	PMT	60	1/w	40	No exercise	①②④⑥⑦
Liu et al., 2018	China	73	63.1 ± 3.3	PMT	60–90	5/w	12	No exercise	①
Liu, 2021	China	34	64.5 ± 4.3	PMT	60	5/w	12	No exercise	②③④
Peng, 2021	China	32	67.3 ± 3.7	PMT	60	3/w	13	Health education	②③④
Sun et al., 2011	China	138	69.2 ± 5.9	PMT	60	2/w	12	No exercise	①
Sun, 2021	China	30	61.4 ± 1.9	PMT	60	3/w	12	No exercise	①②⑤
Xu, 2022	China	30	62.3 ± 1.8	PMT	60	3/w	12	No exercise	②
Zhang et al., 2022	China	60	60–70	PMT	30–60	4–5/w	52	No exercise	①
Miyazaki et al., 2022	Japan	59	67.2	PMT	30	3/week	4	No exercise intervention	②
Mortimer et al., 2012	China	60	67.3 ± 5.3	PMT	50	3/week	40	No exercise intervention	②③
Baklouti et al., 2023	Tunisia	30	64.00 ± 3.02	PMT	60	2/week	100	Stretching exercises	②
García-Garro et al., 2020	Spain	107	69.98 ± 7.83	PMT	60	2/week	12	Balance training	③
Nguyen and Kruse, 2012	Vietnam	73	69.23 ± 5.3	PMT	60	2/week	24	No exercise intervention	③
Lu et al., 2016	China	31	72.8 ± 6.7	PMT	60	3/week	16	No exercise intervention	②
Yang et al., 2020	China	26	66.3 ± 4.9	PMT	45	3/week	8	Health Education	②③④

① Overall cognitive function; ② Inhibitory control; ③ Task-switching ability; ④ Working memory; ⑤ Memory function. RES, resistance training; AER, aerobic exercises; HIIT, high-intensity interval training; PMT, physical and mental training; CEX, multi-modal exercise interventions; CON, control group.

### 3.3 Risk of bias assessment

The methodological quality of the included randomized controlled trials was evaluated using the Cochrane Risk of Bias Tool (RoB 1.0), focusing on five key domains. Each domain was rated as having low risk, some concerns, or high risk of bias. Table 2 provides a summary of the assessment results across all 58 studies. Randomization process was assessed as low risk in 47 studies (81.0%), with 9 studies (15.5%) rated as having some concerns and 2 studies (3.4%) as high risk. Allocation concealment was judged low risk in 36 studies (62.1%), with 15 studies (25.9%) showing some concerns and 7 (12.0%) assessed as high risk. For blinding of outcome assessors, 30 studies (51.7%) were rated as low risk, 18 (31.0%) as some concerns, and 10 (17.3%) as high risk. Missing outcome data was well handled in most studies, with 52 (89.7%) showing low risk, 4 (6.9%) showing some concerns, and 2 (3.4%) as high risk. Selective reporting was evaluated as low risk in 49 studies (84.5%), with 7 (12.1%) having some concerns and 2 (3.4%) showing high risk.

**TABLE 2:** Summary of risk of bias assessment across included studies (*N* = 58).

Domain	Low risk	Some concerns	High risk
1. Randomization process	47 (81.0%)	9 (15.5%)	2 (3.4%)
2. Allocation concealment	36 (62.1%)	15 (25.9%)	7 (12.0%)
3. Blinding of assessors	30 (51.7%)	18 (31.0%)	10 (17.3%)
4. Missing outcome data	52 (89.7%)	4 (6.9%)	2 (3.4%)
5. Selective reporting	49 (84.5%)	7 (12.1%)	2 (3.4%)

Overall, the majority of studies were deemed to have a low risk of bias, though issues related to allocation concealment and blinding were present in a subset of studies. These findings suggest a generally robust evidence base, with moderate risk primarily concentrated in reporting transparency and performance-related domains.

### 3.4 Meta-analysis results

#### 3.4.1 Overall cognitive function

(1) Main effects of different exercise interventions

As shown in Table 3, all four exercise modalities demonstrated significant improvements in overall cognitive function among older adults. Mind-body exercise yielded the greatest effect (SMD = 0.62, 95% CI: 0.38–0.86, *P* < 0.001), followed by resistance training (SMD = 0.55, 95% CI: 0.21–0.89, *P* = 0.002) and aerobic exercise (SMD = 0.49, 95% CI: 0.23–0.75, *P* < 0.001). Multicomponent interventions also showed a significant, though relatively smaller, effect (SMD = 0.38, 95% CI: 0.05–0.71, *P* = 0.030). The SUCRA rankings further supported the superiority of mind-body exercise and resistance training. The optimal protocols for each intervention are summarized in Table 3.

**TABLE 3:** Summary of effects of different exercise interventions on overall cognition.

Intervention type	Effect size (SMD)	95% CI	*P*-value	SUCRA (%)	Optimal protocol
Resistance training	0.55	0.21 to 0.89	0.002	83.3	2/w, 45 min, 12 wk
Aerobic exercise	0.49	0.23 to 0.75	<0.001	68.5	3/w, 60 min, 21 wk
Mind-body exercise	0.62	0.38 to 0.86	<0.001	48.4	3/w, 60 min, 12 wk
Multicomponent	0.38	0.05 to 0.71	0.03	49.3	3/w, 50 min, 16 wk

SMD, standardized mean difference; SUCRA, surface under the cumulative ranking curve, representing the probability of being ranked as the most effective intervention.

(2) Subgroup analyses

Table 4 presents the results of subgroup analyses examining potential sources of heterogeneity. Intervention frequency: High-frequency exercise interventions (≥3 times/week) produced greater cognitive benefits (SMD = 0.63, 95% CI: 0.42–0.84, *P* < 0.001) compared to low-frequency interventions (<3 times/week, SMD = 0.41, 95% CI: 0.12–0.70, *P* = 0.007). Session duration: Sessions of 45–60 min were associated with the largest effect size (SMD = 0.61, 95% CI: 0.40–0.82, *P* < 0.001), while both shorter (<60 min) and longer (> 60 min) sessions also showed significant benefits. Intervention duration: Mid-term interventions (12–24 weeks) yielded the most pronounced effects (SMD = 0.68, 95% CI: 0.46–0.90, *P* < 0.001), followed by long-term (>24 weeks, SMD = 0.55) and short-term interventions (≤12 weeks, SMD = 0.45). Age group: Middle-aged participants (65–75 years) demonstrated the largest cognitive gains (SMD = 0.52, 95% CI: 0.28–0.76, *P* < 0.001), with younger (<65 years, SMD = 0.43) and older ( > 75 years, SMD = 0.48) adults also benefiting. Region: Subgroup analysis by geographic region indicated that studies conducted in Asia reported the greatest effects (SMD = 0.62, 95% CI: 0.40–0.84, *P* < 0.001), followed by Europe (SMD = 0.51) and the Americas (SMD = 0.43). These findings indicate that higher frequency, moderate session duration, and mid-term interventions, as well as interventions targeting Asian populations and those aged 65–75 years, are associated with greater improvements in overall cognitive function.

**TABLE 4:** Subgroup analysis of factors influencing overall cognitive function.

Subgroup variable	Category	Effect size (SMD)	95% confidence interval	*P*-value
Intervention frequency	Low frequency (<3 times/week)	0.41	0.12 to 0.70	0.007
	High frequency (≥3 times/week)	0.63	0.42 to 0.84	<0.001
Session duration	<60 min	0.39	0.11 to 0.67	0.009
	45–60 min	0.61	0.40 to 0.82	<0.001
	>60 min	0.5	0.12 to 0.88	0.01
Intervention duration	Short-term (≤12 weeks)	0.45	0.21 to 0.69	0.001
	Mid-term (12–24 weeks)	0.68	0.46 to 0.90	<0.001
	Long-term (>24 weeks)	0.55	0.18 to 0.92	0.004
Age	Younger (<65 years)	0.43	0.15 to 0.71	0.004
	Middle-aged (65–75 years)	0.52	0.28 to 0.76	<0.001
	Older (>75 years)	0.48	0.20 to 0.76	0.002
Region	Asia	0.62	0.40 to 0.84	<0.001
	Europe	0.51	0.32 to 0.70	<0.001
	Americas	0.43	0.21 to 0.65	0.001

#### 3.4.2 Inhibitory control

As presented in Table 5, all exercise interventions were associated with improvements in inhibitory control among older adults. Mind-body exercise demonstrated the largest effect size (SMD = −0.45, 95% CI: −0.72 to −0.18, *P* < 0.001), followed by HIIT (SMD = −0.35, 95% CI: −0.81 to 0.11, *P* = 0.13), resistance training (SMD = −0.32, 95% CI: −0.65 to −0.01, *P* = 0.04), and aerobic exercise (SMD = −0.28, 95% CI: −0.49 to −0.07, *P* = 0.008). According to SUCRA rankings, resistance training had the highest probability of being the optimal intervention (SUCRA = 82.1%), followed by HIIT (76.1%), mind-body exercise (40.7%), and aerobic exercise (35.1%).

**TABLE 5:** Comparative effects of exercise interventions on inhibitory control.

Exercise type	Effect size (SMD)	95% confidence interval	*P*-value	SUCRA (%)
Resistance training	−0.32	−0.65 to −0.01	0.04	82.1
HIIT	−0.35	−0.81 to 0.11	0.13	76.1
Mind-body exercise	−0.45	−0.72 to −0.18	<0.001	40.7
Aerobic exercise	−0.28	−0.49 to −0.07	0.008	35.1

SMD, standardized mean difference; SUCRA, surface under the cumulative ranking curve, representing the probability of being ranked as the most effective intervention.

Subgroup analyses are summarized in Table 6. High-frequency interventions (≥3 times/week) yielded greater benefits for inhibitory control (SMD = −0.38, 95% CI: −0.61 to −0.15, *P* = 0.001) compared to low-frequency interventions (<3 times/week, SMD = −0.31, 95% CI: −0.52 to −0.10, *P* = 0.003). Sessions lasting 45–60 min showed the largest effects (SMD = −0.36, 95% CI: −0.55 to −0.17, *P* < 0.001), while both shorter and longer sessions had smaller or non-significant effects. Interventions with mid-term duration (12–24 weeks) demonstrated the most pronounced improvement (SMD = −0.41, 95% CI: −0.64 to −0.18, *P* < 0.001), whereas short-term and long-term interventions showed smaller or non- significant effects.

**TABLE 6:** Subgroup analysis of factors influencing inhibitory control.

Subgroup variable	Category	Effect size (SMD)	95% confidence interval	*P*-value
Intervention frequency	Low frequency (<3 times/week)	−0.31	−0.52 to −0.10	0.003
	High frequency (≥3 times/week)	−0.38	−0.61 to −0.15	0.001
Session duration	<60 min	−0.29	−0.56 to −0.02	0.04
	45–60 min	−0.36	−0.55 to −0.17	<0.001
	>60 min	−0.11	−0.98 to 0.76	0.81
Intervention duration	Short-term (≤12 weeks)	−0.3	−0.51 to −0.09	0.005
	Mid-term (12–24 weeks)	−0.41	−0.64 to −0.18	<0.001
	Long-term (>24 weeks)	−0.22	−0.89 to 0.45	0.51
Age	Younger (<65 years)	−0.25	−0.68 to 0.18	0.25
	Middle-aged (65–75 years)	−0.36	−0.55 to −0.17	<0.001
	Older (>75 years)	−0.31	−0.74 to 0.13	0.17
Region	Asia	−0.48	−0.79 to −0.17	0.003
	Europe	−0.3	−0.50 to −0.10	0.003
	Americas	−0.27	−0.59 to 0.05	0.09

Regarding age, the most significant improvements were observed in the 65–75 years subgroup (SMD = −0.36, 95% CI: −0.55 to −0.17, *P* < 0.001), while younger and older participants did not show statistically significant benefits. Regionally, studies conducted in Asia reported the greatest improvements (SMD = −0.48, 95% CI: −0.79 to −0.17, *P* = 0.003), followed by Europe and the Americas.

Collectively, these findings indicate that resistance training and mind-body exercise, especially when delivered at moderate frequency (≥3 times/week) and duration (45–60 min/session, 12–24 weeks), are particularly effective for enhancing inhibitory control in older adults. The greatest benefits appear to occur among participants aged 65–75 years and in Asian populations.

#### 3.4.3 Task-switching ability

As shown in Table 7, mind-body exercise produced the most substantial improvement in task-switching ability among older adults (SMD = −0.58, 95% CI: −0.89 to −0.27, *P* < 0.001; SUCRA = 85.1%), with statistically significant benefits compared to other interventions. Resistance training (SMD = −0.21, 95% CI: −0.45 to 0.03, *P* = 0.08; SUCRA = 51.4%), aerobic exercise (SMD = −0.18, 95% CI: −0.47 to 0.11, *P* = 0.23; SUCRA = 40.3%), and HIIT (SMD = −0.35, 95% CI: −0.81 to 0.11, *P* = 0.13; SUCRA = 39.2%) also showed trends toward improvement, but did not reach statistical significance.

**TABLE 7:** Comparative effects of exercise interventions on task-switching ability.

Exercise type	Effect size (SMD)	95% Confidence interval	*P* value	SUCRA (%)
Mind-body exercise	−0.58	−0.89 to −0.27	<0.001	85.1
Resistance training	−0.21	−0.45 to 0.03	0.08	51.4
Aerobic exercise	−0.18	−0.47 to 0.11	0.23	40.3
HIIT	−0.35	−0.81 to 0.11	0.13	39.2

SMD, standardized mean difference; SUCRA, surface under the cumulative ranking curve, reflecting the likelihood of being ranked as the most effective intervention.

Subgroup analyses are presented in Table 8. Higher intervention frequency (≥3 times/week) was associated with greater improvements in task-switching ability (SMD = −0.40, 95% CI: −0.78 to −0.02, *P* = 0.04), compared to low frequency (<3 times/week, SMD = −0.32, 95% CI: −0.54 to −0.10, *P* = 0.004). Sessions of 45–60 min resulted in the largest effects (SMD = −0.39, 95% CI: −0.64 to −0.14, *P* = 0.002), while both shorter and longer sessions were less effective or not statistically significant. Mid-term interventions (12–24 weeks) yielded the most pronounced benefits (SMD = −0.43, 95% CI: −0.72 to −0.14, *P* = 0.004), whereas short-term and long-term durations showed weaker or non-significant effects. With respect to age, middle-aged participants (65–75 years) experienced the greatest gains (SMD = −0.37, 95% CI: −0.58 to −0.16, *P* < 0.001), while younger (<65 years) and older (> 75 years) groups did not achieve significant improvements. Regionally, studies conducted in Asia reported the largest effects (SMD = −0.51, 95% CI: −0.82 to −0.20, *P* = 0.001), compared to Europe and the Americas.

**TABLE 8:** Subgroup analysis of factors influencing inhibitory control.

Subgroup variable	Category	Effect size (SMD)	95% confidence interval	*P*-value
Exercise type	Resistance training	−0.21	−0.45 to 0.03	0.08
	Aerobic exercise	−0.18	−0.47 to 0.11	0.23
	Mind-body exercise	−0.58	−0.89 to −0.27	<0.001
	HIIT	−0.34	−1.13 to 0.45	0.41
Intervention Frequency	Low frequency (<3 times/week)	−0.32	−0.54 to −0.10	0.004
	High frequency (≥3 times/week)	−0.4	−0.78 to −0.02	0.04
Session Duration	<60 min	−0.25	−0.52 to 0.02	0.07
	45–60 min	−0.39	−0.64 to −0.14	0.002
	>60 min	−0.34	−0.76 to 0.08	0.11
Intervention Duration	Short-term (≤12 weeks)	−0.31	−0.55 to −0.07	0.01
	Mid-term (12–24 weeks)	−0.43	−0.72 to −0.14	0.004
	Long-term (>24 weeks)	−0.03	−0.65 to 0.59	0.92
Age	Younger (<65 years)	−0.19	−0.81 to 0.43	0.54
	Middle-aged (65–75 years)	−0.37	−0.58 to −0.16	<0.001
	Older (>75 years)	−0.28	−1.23 to 0.67	0.57
Region	Asia	−0.51	−0.82 to −0.20	0.001
	Europe	−0.24	−0.48 to 0.00	0.05
	Americas	−0.31	−0.70 to 0.08	0.12

In summary, mind-body exercise, particularly when delivered at moderate frequency (≥3 times/week), with session durations of 45–60 min and intervention periods of 12–24 weeks, is especially effective for enhancing task-switching ability in older adults. Middle-aged participants and those in Asian settings appear to benefit the most from these interventions.

#### 3.4.4 Working memory

As presented in Table 9, both aerobic exercise (SMD = 0.58, 95% CI: 0.12 to 1.04, *P* = 0.01) and mind-body exercise (SMD = 2.45, 95% CI: 1.48 to 3.42, *P* < 0.001) significantly improved working memory among older adults, with mind-body exercise demonstrating a notably larger effect size.

**TABLE 9:** Subgroup analysis of factors influencing working memory.

Subgroup variable	Category	Effect size (SMD)	95% Confidence interval	*P*-value
Exercise type	Aerobic exercise	0.58	0.12 to 1.04	0.01
	Mind-body exercise	2.45	1.48 to 3.42	<0.001
Intervention frequency	Low frequency (<3 times/week)	1.32	−0.15 to 2.79	0.08
	High frequency (≥3 times/week)	1.68	0.89 to 2.47	<0.001
Session duration	45–60 min	1.89	1.12 to 2.66	<0.001
Intervention duration	Short-term (≤12 weeks)	1.72	0.45 to 2.99	0.007
	Mid-term (12–24 weeks)	1.05	−0.32 to 2.42	0.13
Age	Middle-aged (65–75 years)	1.86	1.09 to 2.63	<0.001
Region	Asia	2.23	1.26 to 3.20	<0.001
	Europe	0.48	−0.07 to 1.03	0.08

Subgroup analyses (Table 9) showed that high-frequency interventions (≥3 times/week) yielded greater improvements in working memory (SMD = 1.68, 95% CI: 0.89 to 2.47, *P* < 0.001) than low-frequency interventions. Sessions of 45–60 min (SMD = 1.89, 95% CI: 1.12 to 2.66, *P* < 0.001) and short-term interventions (≤ 12 weeks, SMD = 1.72, 95% CI: 0.45 to 2.99, *P* = 0.007) were associated with the most pronounced benefits. Middle-aged participants (65–75 years) experienced the greatest improvements (SMD = 1.86, 95% CI: 1.09 to 2.63, *P* < 0.001). Regional analysis indicated that interventions conducted in Asia produced the largest effect (SMD = 2.23, 95% CI: 1.26 to 3.20, *P* < 0.001), while studies from Europe showed a smaller and non-significant improvement.

In summary, mind-body exercise, particularly when delivered at high frequency, with moderate session duration and short-term intervention period, is especially effective for enhancing working memory in older adults, with middle-aged individuals and Asian populations benefiting the most.

#### 3.4.5 Memory function

As shown in Table 10, all exercise interventions produced positive effects on memory function among older adults. Mind-body exercise exhibited the greatest effect size (SMD = 0.58, 95% CI: 0.34 to 0.82, *P* < 0.001), followed by aerobic exercise (SMD = 0.42, 95% CI: 0.23 to 0.61, *P* < 0.001), resistance training (SMD = 0.35, 95% CI: 0.12 to 0.58, *P* = 0.003), and multicomponent exercise (SMD = 0.28, 95% CI: −0.02 to 0.58, *P* = 0.07), with the last not reaching statistical significance.

**TABLE 10:** Comparative effects of exercise interventions on memory function.

Subgroup variable	Category	Effect size (SMD)	95% Confidence interval	*P*-value
Exercise type	Resistance training	0.35	0.12 to 0.58	0.003
	Aerobic exercise	0.42	0.23 to 0.61	<0.001
	Mind-body exercise	0.58	0.34 to 0.82	<0.001
	Multicomponent exercise	0.28	−0.02 to 0.58	0.07
Intervention frequency	Low frequency (<3 times/week)	0.32	0.11 to 0.53	0.003
	High frequency (≥3 times/week)	0.51	0.32 to 0.70	<0.001
Session duration	<60 min	0.38	0.15 to 0.61	0.001
	45–60 min	0.49	0.31 to 0.67	<0.001
	>60 min	0.3	−0.12 to 0.72	0.16
Intervention duration	Short-term (≤12 weeks)	0.36	0.17 to 0.55	<0.001
	Mid-term (12–24 weeks)	0.53	0.32 to 0.74	<0.001
	Long-term (>24 weeks)	0.48	0.11 to 0.85	0.01
Age	Younger (<65 years)	0.31	0.03 to 0.59	0.03
	Middle-aged (65–75 years)	0.46	0.30 to 0.62	<0.001
	Older (>75 years)	0.39	0.12 to 0.66	0.006
Region	Asia	0.62	0.38 to 0.86	<0.001
	Europe	0.38	0.21 to 0.55	<0.001
	Americas	0.41	0.18 to 0.64	0.001

Subgroup analyses (Table 10) revealed that high-frequency interventions (≥3 times/week, SMD = 0.51, 95% CI: 0.32 to 0.70, *P* < 0.001), moderate session duration (45–60 min, SMD = 0.49, 95% CI: 0.31 to 0.67, *P* < 0.001), and mid-term intervention duration (12–24 weeks, SMD = 0.53, 95% CI: 0.32 to 0.74, *P* < 0.001) produced the largest improvements in memory function. All age groups benefited, with the most pronounced effect in middle-aged participants (65–75 years, SMD = 0.46, 95% CI: 0.30 to 0.62, *P* < 0.001). Regional analysis showed that studies conducted in Asia reported the greatest improvement (SMD = 0.62, 95% CI: 0.38 to 0.86, *P* < 0.001), followed by the Americas and Europe.

In summary, mind-body exercise, especially when delivered with high frequency, moderate session duration, and mid-term intervention period, is particularly effective for improving memory function in older adults, with the most substantial benefits observed in middle-aged individuals and Asian populations.

## 4 Discussion

Cognitive decline, including mild cognitive impairment and dementia, is a critical challenge in global aging (Petersen et al., 2014b; Liu J. et al., 2023). Our network meta-analysis systematically compared five exercise modalities across multiple cognitive domains in cognitively healthy older adults, incorporating a significant number of Chinese RCTs to improve geographic representativeness and reveal population-specific effects. This study identified not only the optimal exercise protocols for different cognitive domains but also highlighted protocol- and population-specific patterns that advance the precision of exercise prescription in aging societies.

### 4.1 Impact of resistance training on cognitive function

Our network meta-analysis found that resistance training was the most effective intervention for improving global cognitive function in cognitively healthy older adults, with a pooled effect size of SMD = 0.55. In addition, the effect on inhibitory control was also the most pronounced among all exercise modalities (SMD = 0.31, SUCRA = 82.1%). Notably, our subgroup analysis indicated that the optimal protocol consisted of 12 weeks of resistance training, performed twice per week, with each session lasting 45 min, which produced the largest and most significant cognitive gains. These benefits were particularly evident in participants aged 65–75 years and in studies conducted in Asian regions. Our findings are consistent with, but in some cases exceed, those reported in previous meta-analyses. For example, Barha et al. (2017) found that resistance training improved global cognition (SMD = 0.22, 95% CI: 0.09–0.36) and executive function in older adults, while Northey et al. (2018) observed effect sizes of SMD = 0.13–0.22 for resistance-based interventions in adults over 50. The larger effect size in our analysis may be attributed to differences in participant characteristics, higher adherence rates, or optimized training protocols in the included studies, especially those from East Asia.

Mechanistically, the cognitive benefits of resistance training are supported by substantial neurobiological evidence. Resistance training has been shown to elevate brain-derived neurotrophic factor (BDNF) levels, which supports synaptic plasticity and hippocampal neurogenesis, both critical for cognitive health (Schley et al., 2006; Loprinzi et al., 2019). Coelho et al. (2012) demonstrated that a 10-week program of lower limb resistance training increased plasma BDNF by 65.2% in older participants, which is linked to improved memory and reduced age-related neurodegeneration. Additional mechanisms may include stimulation of vascular endothelial growth factor, growth hormone, and IGF-1, as well as reductions in inflammatory cytokines (Engeroff et al., 2022). Taken together, our results support a clear, evidence-based recommendation for resistance training (twice weekly, 45 min per session, for at least 12 weeks) as the optimal protocol to enhance global cognition and inhibitory control in older adults. This approach is accessible, cost-effective, and consistent with WHO guidelines (Ahlskog et al., 2011), and should be considered as a key component of cognitive health promotion strategies in aging populations.

### 4.2 Impact of exercise interventions on executive function

In our network meta-analysis, mind-body exercise (e.g., Tai Chi, Baduanjin) demonstrated the most significant benefits for executive function, particularly in task-switching ability (SMD = −0.58, SUCRA = 85.1%) and working memory (SMD = 2.45). Resistance training also produced clear improvements in inhibitory control (SMD = 0.31, SUCRA = 82.1%), while aerobic exercise contributed moderate but positive effects on executive domains. Our subgroup analyses further revealed that the optimal protocol for executive function improvement involved high-frequency interventions (≥3 times/week), moderate session duration (45–60 min), and short- to mid-term durations (12–24 weeks). The greatest cognitive benefits were observed in participants aged 65–75 years and in Asian populations.

These results are consistent with, and in some cases extend, previous literature: Gheysen et al. (2018) performed a meta-analysis and reported that mind-body exercises led to moderate improvements in cognitive flexibility and working memory (Hedges’ g = 0.36, 95% CI: 0.15–0.58). Our results suggest a larger effect size, which may reflect differences in population structure, cultural familiarity with mind-body practices, or higher adherence in the included Asian studies. Barha et al. (2017) identified that resistance training improved executive function in older adults (SMD = 0.22, 95% CI: 0.09–0.36), consistent with our findings for inhibitory control. Northey et al. (2018) reported that combined and aerobic exercise programs also benefit executive function (SMD ≈ 0.14–0.20), in line with our observation of positive, though comparatively smaller, effects for aerobic interventions. Possible mechanisms underlying these improvements include enhanced activation and plasticity of the prefrontal cortex, upregulation of neurotrophic factors (such as BDNF), improved cerebrovascular function, and reductions in stress-related hormones (Colcombe and Kramer, 2003; Kramer and Colcombe, 2018). Mind-body exercise, with its emphasis on attentional control, sequencing, and body awareness, may optimally stimulate neural circuits responsible for executive processing.

In summary, our data support prioritizing mind-body exercise (≥3 times/week, 45–60 min/session, 12–24 weeks) as the most effective intervention for enhancing executive function, especially task-switching and working memory, in older adults. Resistance training remains the preferred approach for improving inhibitory control, and both modalities can be integrated for broader executive benefits.

### 4.3 Impact of exercise interventions on memory function

Our network meta-analysis revealed that mind-body exercise exhibited the largest effect on memory function (SMD = 0.58), followed by aerobic exercise (SMD = 0.42) and resistance training (SMD = 0.35) among healthy older adults. The optimal protocol for memory improvement was characterized by high-frequency intervention (≥3 times/week), moderate session duration (45–60 min), and mid-term intervention duration (12–24 weeks). The greatest memory benefits were observed in participants aged 65–75 years and in Asian regions.

These findings are consistent with previous high-quality reviews and meta-analyses: Zheng et al. (2023) reported in a large-scale network meta-analysis that mind-body exercise and aerobic training significantly improved memory performance (SMD = 0.39 and 0.31, respectively), closely matching the effect sizes observed in our study. Blondell et al. (2014) concluded that aerobic interventions of at least 24 weeks’ duration produced more pronounced memory gains (mean difference: 0.41), reinforcing our subgroup results that longer, sustained exercise is particularly effective for memory enhancement. Smith et al. (2010) found a smaller effect size for aerobic interventions (SMD = 0.13 for memory), which may be due to differences in intervention intensity, sample characteristics, or adherence.

Neurobiological mechanisms underpinning these benefits include increased hippocampal neurogenesis and synaptic plasticity, mediated by exercise-induced elevation of BDNF and IGF-1, improved cerebrovascular function, and reductions in chronic inflammation (Tao et al., 2019; Riske et al., 2017; Liu J. et al., 2023). Mind-body exercise may further enhance memory through stress reduction, attention regulation, and improved executive control, all of which support memory encoding and retrieval. Taken together, our results recommend mind-body and aerobic exercise—particularly high-frequency, moderate-duration, mid-term interventions—for the enhancement of memory function in older adults. These findings support the integration of structured exercise programs into aging and dementia prevention strategies, with specific emphasis on Asian populations and the middle-aged older who demonstrated the most robust benefits.

### 4.4 Implications for public health and clinical practice

The results of our network meta-analysis provide a strong foundation for precision exercise prescriptions to promote cognitive health in older adults. By identifying the optimal exercise modality and protocol for each cognitive domain, our findings support the development of individualized intervention strategies based on specific cognitive goals and baseline characteristics. Resistance training is recommended as the primary intervention for enhancing overall cognitive function and inhibitory control. Mind-body exercise (e.g., Tai Chi, Baduanjin) is most effective for improving working memory and task-switching ability, making it suitable for those seeking to boost executive function. Aerobic exercise should be prioritized for individuals targeting memory enhancement.

For sedentary but cognitively healthy older adults, community-based group programs that combine aerobic and resistance training (e.g., 20 min of brisk walking plus 15 min of bodyweight exercises) can maximize cognitive, physical, and social benefits. For those with mobility limitations, low-intensity, seated mind-body exercises (such as chair Tai Chi or adaptive yoga) provide accessible means to improve balance, executive function, and engagement without excessive physical stress.

Widespread promotion of these evidence-based exercise interventions could yield significant public health gains—not only by delaying cognitive decline and reducing dementia risk, but also by lowering healthcare costs and enhancing quality of life (Blondell et al., 2014; Norton et al., 2014). Public health campaigns should highlight the cognitive benefits of regular physical activity, integrating this message into routine health promotion for older adults. Crucially, while our findings underscore the effectiveness of specific protocols, long-term adherence and embedding exercise into daily life are essential for consolidating cognitive benefits and achieving sustainable impact. Moving forward, clinical guidelines and public health initiatives should emphasize personalized, sustainable exercise routines that are tailored to individual health status, functional capacity, and cognitive goals. This precision approach will maximize benefit at both the individual and societal level in the context of global population aging.

### 4.5 Study limitations and future directions

Despite the strengths of this study, several limitations should be acknowledged. First, the variability in cognitive assessment tools (e.g., MoCA, Stroop Test) across studies may limit comparability. Future research should aim to standardize cognitive outcome measures to facilitate more consistent comparisons. Additionally, this study primarily focused on cognitive function, executive function, and memory, without considering other cognitive domains such as attention and language fluency. Future studies should broaden their scope to include these domains, offering a more comprehensive understanding of how exercise influences cognitive health. Finally, while this study explored the effects of various exercise modalities, the intensity of these interventions was not examined in detail. Future research should investigate how varying exercise intensities affect cognitive outcomes, with the goal of developing more refined exercise prescriptions for older adults.

## 5 Conclusion

In summary, this network meta-analysis provides robust evidence that different exercise modalities yield domain-specific cognitive benefits in cognitively healthy older adults. Resistance training was identified as the most effective intervention for improving global cognitive function and inhibitory control, particularly when delivered as twice-weekly sessions of 45 min over 12 weeks. Mind-body exercise demonstrated the greatest improvements in executive function, including task-switching ability and working memory, especially with high-frequency, moderate-duration protocols. Aerobic exercise was most effective for enhancing memory function, with sustained benefits observed in mid- to long-term interventions.

Our results also highlight that personalized exercise prescriptions, tailored to individual cognitive needs and functional capacity, should be prioritized in both clinical and public health contexts. The cognitive gains were most pronounced in middle-aged older adults (65–75 years) and among Asian populations, suggesting the need for culturally and demographically sensitive interventions.

## Data Availability

The original contributions presented in this study are included in this article/Supplementary material, further inquiries can be directed to the corresponding author.
