# Supplementary material for: Optimal exercise interventions for enhancing cognitive function in older adults: a network meta-analysis
Source: Front Aging Neurosci. 2025 Jul 11;17:1510773. doi: 10.3389/fnagi.2025.1510773 (PMC12289702; doi:10.3389/fnagi.2025.1510773)
Supplement: Supplementary file 1 [file Table_1.docx]

**Supplementary Material 1: Search Strategy**

We systematically searched several major databases, including PubMed, Web of Science, ScienceDirect, CNKI, and Wanfang Data, for randomized controlled trials (RCTs) evaluating the effects of exercise interventions on cognitive function in healthy older adults. The search was conducted using the following search terms, which were combined to ensure comprehensive coverage:

- ("exercise" OR "physical activity") AND ("cognitive function" OR "cognition") AND ("older adults" OR "elderly") AND ("memory" OR "executive function") AND ("randomized controlled trial" OR "RCT") AND ("healthy aging").

Searches included both English and Chinese publications and covered all articles published before January 1, 2024. Additionally, reference lists from relevant systematic reviews and meta-analyses were manually screened to identify additional studies, including those by Gallardo-Gomez et al., 2022, and Gavelin et al., 2021.

The complete search strategy is detailed above and can be accessed in this supplementary material.
